# 

**Published:** 1799-07

**Authors:** 


### *Liſt of New Medical and Phyſical Publications in June.*

Memoirs of Medicine, including a sketch of Medical hiſtory from the earlieſt accounts to the eighteenth century:—By RICHARD WALKER, Esq. Apothecary to the Prince of Wales.—London, (price 5s.) Johnson.

A Table of Symptoms, pointing out such as distinguish one disease from another, as well as those which shew the degree of danger in each disease; to which are added, Observations on the excessive indulgence of children, &c.—By J. PARKINSON, London.

An Essay on the most rational means of preserving health: 112 pp. 8vo. London, WALLIS.

Tracts and Observations on Natural History and Physiology. By ROBERT TOWNSON, LL.D. 8vo. 7s. Boards.

Essays on Electricity. By the late E. W. ADAMS. 5th edition, corrected by W. JONES. 1 vol. 8vo. 8s. boards. W. &. S. Jones, *Holborn.*

### NEW MEDICAL PUBLICATIONS IN FRANCE.

*Medecine preservative et curative*;—On the prevention and cure of diseases by medicine, &c. By N. F. ROUGNON. 2 vol. 8vo. 500 pp. *Paris.* Mequignon.

*Tableaux comparatifs de l'anatomie, &c.* Comparative views of the anatomy of domestic animals used in agriculture, &c. By J. GIRARD. *Paris.* Huzard.

*Observations sur un ecoulement spermatique involuntaire dans un cheval.* On a case of an involuntary spermatic running in horses. By C. HUZARD. 8vo. *Paris.*

---

### TO OUR CORRESPONDENTS.

*It has always been conſidered by us, as an object of the firſt importance, in eſtabliſhing this Journal, to hold out the ſtrongeſt inducements to reſpectable practitioners, to diſtinguiſh our publication by valuable original communications. Theſe inducements can only depend on the promptitude and correctneſs with which we exhibit their lucubrations. The* former *we ſhall, in general, be able to command, if our correſpondents will have the goodneſs to recollect the inſertion of the* date *in their letters; but the* latter *can alone be ſecured by their recollecting the* data. *The correct Orthography of Proper Names of Perſons and Places; the deſignations of Rank and Profeſſions, as well as Quotations from Foreign Languages, we requeſt them to tranſmit to us in the moſt legible and unequivocal manner.*

---

### ERRATA.

No. III. p. 221, l. 24, for "antris ferialibus," read "antris *feralibus*"

No. III. p. 273, in the note, for "1764" read, 1794.

No. IV. p. 332, l. 13, from the bottom for "finding" read, "*fixing* the globe of the eye.

No. IV. p. 352, l. 18. for "acid fæces," *acrid* fæces."

————ib. in the note: for "to be a part," read, "to be a *nice* part",

---

END OF VOL. I.

### INDEX.

ABICH, M. on the honey-ſtone 398

Abilgaart, Prof. his diſcovery of pot-aſh in animal blood 300

Academy of ſurgery, at Copenhagen 399

Accum, Mr. his "Chemico-Phyſical Aphoriſms" - 90

Achard, Mr. of Berlin, his experiments in electricity 204

Acid, oxygenated muriatic, combination of, with ammoniac 300

Addreſs to the public 312

Air, atmoſpherical, obſervations on 27

"*Albini Tabulæ Anatomicæ*," &c. advertiſed for ſale 298

Allouel, citz. his "*Surgical Terms*," 415

Alyon, citz. his "*Elementary View of Chemiſtry*" 211

American Mineralogical Society 86

Anderſon, Dr. on the preſervation of apples from froſt 297

Animation, ſuſpended, uſe of emetics in 59

Anſwers to correſpondents 416

Apples, means of preſerving them from froſt, by Dr. Anderſon 297

Archer, Dr. his remedy in croup, or cynanche trachealis 83

———, Dr. Thomas, of Maryland, on a caſe of difficult parturition, ſucceſsfully treated 157

———, Dr. his "Inaugural Diſſertation on Cynanche Trachealis" 106

Arnemann, Dr. on the amauroſis, or gutta ſerena 24

———, Dr. his "Practical Syſtem of the Materia Medica" 99

Aſphyxia in children, new method of preventing 182

Ayerer, Dr. on a new inſtrument for lithotomy 302

Baillie, Dr. Matthew, his retirement ibid.

Bark, Peruvian, ſubſtitutes for, in intermittents 190

Barker, Dr. I. on the virtues of lime, magneſia, &c. in fevers 274

Barthez's analyſis of the mechaniſm of motion in men and animals 97

Barton, Dr. his "Collections," &c. 99

Baudeloque, citz. on the Cæſarian operation 302

Baumé, citz. on the decompoſition of the calcareous muriat of lime 197

———, citz. his "*Chemical Tracts*" 212

Bayen, Pierre, his works publiſhed by citizen Malatret 200

Beckmann, Prof. his experiments on ſtaining wood 204

Beddoes, Dr. his "Contributions to Phyſical and Medical Knowledge" 89

———, Dr. his "Collection of Teſtimonies" 100

———, Dr. his notice of erratum 144

Beddoes, Dr. correction of an erratum in his "*Weſt Country Contributions*" 243

———, Dr. his "*Eſſay on Conſumption*" 410

——— on Fourcroy's expoſition 514

Bell, Mr. C. his ſyſtem of diſſections 403

———, Mr. B. remarks on his ſyſtem of ſurgery 517

———, Mr. J. remarks on his anatomy ib.

Berthollet, citz. on the acid obtained from diſtilled animal ſubſtances 85

Blair, Mr. his "*Eſſays on the Venereal Diſeaſe*" 409

Books, new, notices of 110

Boſtock, Dr. on the nitrat of ſilver in epilepſy 222

Bouſſey's "Eſſay on intermitting Fevers," &c. 104

Bourguet's "Elements of Natural Philoſophy" 98

Boutrolle's "Complete Herdſman" 107

Bradley, Dr. his account of the cow-pox 1

———, Dr. his lectures 302

———, Dr. commencement of his lectures 403

Brera, on medicated frictions 71

———, Prof. his annunciation of "*Ratio Medendi*," &c. 203

———, ——— his reſignation 402

———, ——— — "*Table of Diſeaſes*" 411

———, ——— — "*Reflections on the Uſe of Phoſphorus*" 412

———, ——— — "*Memoir on the Cat-Diſtemper*" 412

Brickell, Dr. on the puerperal fever 381

Brieude, citz. J. C. on the odours exhaled from the body 243

Britiſh Mineralogical Society 404

Brouſſonet, citz. his account of the proceſs for making morocco of different colours 201

Brown, Mr. Charles, his "Annals of Pneumatic Medicine" 89

Browne's "Treatiſe on the Yellow Fever" 106

Brown, Mr. C. his account of a caſe of apoplexy 331

———, Mr. on a ſingular obſtruction in the ureters 449

Bruſe, Mr. his patent inſtruments for "extracting teeth in a perpendicular direction" 87

Brugnatelli, citz. his concurrence with Fourcroy and Vauquelin 85

———, citz. on light 199

———, on fulminations by phoſphorus, &c. ibid.

Brunonian ſyſtem, account of the 124

Calculi, biliary, remedy for 394

———, ſtone and gravel, on the ſucceſsful treatment of 507

Cæſarean operation, propriety of performing the 391

Cam, Mr. on the uſe of the trephine 328

Caries ossium, remarks on, by Dr. Lentin 294

Carlisle, Mr. his new method of applying the tourniquet 23

———, Mr. A. his lectures 90

———, Mr. his account of an instrument for cutting the cornea 332

Carradori, Dr. on the new system of chemistry 198

Carrere, citz. his catalogue of works on mineral waters 305

———, citz. his "*Treatise on the Properties, &c. of the Solanum Scandens*" 307

Castel, citz. his "*Poem on Plants*" 209

Cavanilles, D. A. his description of the wild plants of Spain 210

Chancres, venereal, cured by alkalies 189

Chapman, Dr. Isaac, on a species of cantharis 143

Chaptal, citz. J. A. on the effect of mordicants, in dying cotton red 168

———, citz. his chemical observations on the epidermis 244

Children, on bleeding those with large heads, 390

———, apoplectic cases of 392

Christie, Mr. on dysentery 347, 463

Clarke, Dr. his "Medicinæ Traxeos Compendium" 90

———, Dr. E. G. his work, entitled Medicinæ Traxeous Compendium 308

———, Richard, M. D. his "*Medical Strictures ! !*" 308

Collier, Mr. his patent for a chemical process for freeing fish oils from their impurities, &c. 86

———, Mr. his new machines for purifying water 402

Collin, Dr. on sphacelus 510

Collomb's "Medical and Chirurgical Essays," &c. 104

Comparative anatomy, by citz. Cuvier, now in the press 299

Conradi, Dr. his practical remarks on the cramp in the stomach 51

Coppen, Mr. on the decomposition of sea-salt 301

Correspondents, answers to 311

Cornelli, Mr. on the distillation of vinegar, 80

Costiveness, use of carbon in 393

Courset, citz. on plants 400

Cow-pox, history of the inoculation for the 217

———, history of inoculation for the, continued 313

Currie, Dr. his "Observations on Fevers" 105

———, Dr. W. on the yellow fever 213

———, — on the causes of bilious fever 246

Custance, Mr. on a case of monstrosity 451

Cuvier's "Elementary Picture of the Natural History of Animals" 97

———, citz. his "*Natural History of Animals*," &c. 208

Cyrilli, Dominique, "*On the scarce Plants of Naples*" 210

Davidge, Dr. his "Treatise on the Autumnal Epidemic," &c. 105

Davies, Dr. on a remarkable case of petechiæ

Deaths, list of, in London, for the last three months 336

De Candole, citz. his description of the *Sennebieria pinnatifida* 304

Desgranges, on mineral acids ib.

Delarive, Dr. G. C. on the disease termed chorea sancti viti 21

De Loys, his "*History of Natural Philosophy*" 212

Delile, citz. on Egyptian plants 400

Delunel, citz. on opium 398

De Mertens, Dr. his "*Account of the Plague at Moscow*" 411

Default, citz. on the "*Diseases of the Urinary Passages*" 412

Desaussure, H. B. of Geneva, death of 206

Desfontaines, citz. his "Flora Atlantica" 97

———, citz. his publication of the Flora of Mount Atlas 201

Desgranges, Dr. on the poisonous effects of mineral acids 508

Desessartz, citz. his treatise on the physical education of children 310

Dewees, Dr. his "*Observations on the Use of the Warm Bath in laborious Parturition*" 288

De Witt, Dr. on the effects of the datura stramonium 84

———, his account of the mineral and medical waters of New-York 86

Diabetes mellitus, case of the 218

Digitalis, further remarks on the 383

Diseases, monthly reports of 110

——— of London, general remarks on the prevailing 35, 233

———, list of, in the practice of a physician at the west end of London, 136

———, monthly, account of, in the eastern district of London 215

———, state of, in London 334

Dissertations, inaugural, at Leipzig 402

Drake, Dr N. on the digitalis in the cure of consumption 290

Dropsy, singular case of, after the itch, cured by sulphur 195

Dufresnoy, citz. A. on the nature and cure of ring-worms, &c. 308

Duncan, sen. and jun. their "*Annals of Medicine*" 413

Durande, on biliary calculi 507

Dwight, Dr. N. on the sick head-ach 286

Dyce, Dr. his remarks on the extraction of teeth 13

Dysentery, Mr. Christie on 347

Economical Society of Batavia, their prize-question 296

Eneula helenium, properties of the 298

English medical books translated into German, account of 88

Epilepsy, medicines employed in 182

Errata in Number II. 216

——— III. 311

——— III. and IV. 416, 518

Eudiometer, essays on the, by citz. Jurine and Gattoni 396

Eustis, Dr. his letter on cold air and water in fevers 18

Fabas, citz. on mineral springs 414
Fabroni, Mr. on alcohol 301
Fauft, Dr. his circular letter on the small-pox 83
Fever, yellow, remarks on 78
——, puerperal, its treatment 386
Fontenille, citz. his "*Syftem of Botany*" 209
Forfyth, Mr. on the deftruction of a new fpecies of orchard infect 202
——, Mr. addition to his recipe for deftroying infects on fruit-trees 304
Fourcroy, citz. extract from his effay "On the application of pneumatic chemiftry to the practice of medicine" 82
—— on the application of pneumatic chemiftry to the cure of difeafes 145
—— and Vauquelin, citizens, on urine 197
——, on the experiments of Mayow 198
—— his obfervations on Mayow 249
Fowler, Dr. R. on the cure of confumption by the digitalis 291
Frank, Dr. on the theories of chemiftry 63
—— his "Treatife on the Method of rearing healthy Children" 107
—— Mr. on the Brunonian fyftem 131
—— fen. Dr. on the theories of chemiftry 170
—— Mr. John, annunciation of his "*Obfervations on Typhus-contagion,*" &c. 205
——, Dr. of Vienna, on the theories of chemiftry 269
——, Dr. on the theories of chemiftry 368
*Fritillaria Regia, Linn.* means of multiplying it 297
Fruit-trees, means of preferving their bloffoms from damage by froft 297

Galvanifm, remarks on the practical utility of 53

Gangrene and fphacelus 509

Gefenius, M. Collin 328

——, Dr. on phofphorus 283

Gafolitre, citz. Goldfchmidt, on the 365

Gavard, H his "*Treatife on Mythology*" 306

Gehler, his abridgement of "The Phyfical Dictionary" 90

Gilbert, citz. on modern medical theories 360

—— on the new medical theories 449

—— his "*Hiftory of European Plants* 210

Goettling, Prof. on procuring atmofpheric air 299

Goldfchmidt, citz. on factitious mineral waters 296

Gonorrhœa, new remedy againft it 512

Gruber, Dr. his tranflation of Gren's chemiftry 514

Gregorio, Don M. H. de, his "*Elementary Dictionary of Pharmacy*" 307

Gren, Prof. F. A. C. death of 206

Halle, Jurine, Gattoni, citz. on the eudiometer 397
Head, the probable fenfations of the, after being fevered from the body 51
Health, Society of, their tranfactions 110
——, effay on preferving 498
Hebert, M. his method of making vinegar 516
Helle, Prof. his experiments on phofphorus 300
Henderfon, Mr. on the prefervation of the health of feamen 137
Henderfon, Mr. on the health of feamen, continued 340
——, his account of difeafes on board the Aftrea 236
——, his mode of preferving the health of feamen 91
——, on the difeafes of the Cape of Good Hope 455
Herbell, Mr. J. F. M. his work on anatomy 305
Hermbftaedt, Dr. on the ftudy of chemical botany 73
——, Dr. on the ftudy of chemical botany 159
——, Dr. on the ftudy of chemical botany 263
——, Dr. on the ftudy of chemical botany, continued 377
——, Dr. on chemical botany 502
Hildebrant, M. on preparing the oxyd of mercury 516
Hoffman, Dr. of Mayence, on gangrene and fphacelus 510
Holyoke, Dr. on a new manner of preparing the *fal aëratas* 295
Horfefield's "Experimental Differtation on the Rhus Vernex," &c. 108
Hofpice de l'Unité, defcription of 81
—— l'Humanité, at Paris, defcription of 80
Hufeland, Prof. on the Brunonian fyftem 41
——, on narcotic remedies in inflammations of the eyes 284
——, on a cafe of cancer 285
—— and Kant, on the art of prolonging life 259
Hughes, Mr. his letter to Dr. Jenner 318
Hull, Dr. on the Cæfarean operation 517
Humboldt, Von, on the abforption of oxygen 302
Hutter, J. C. his publication of the "Flora Americana Septentrionalis" 201
Hydrargyrum phofphoratum, a powerful remedy in fyphilis 280

Jackfon, Dr. R. his "*Hiftory and Cure of Fever*" 214
——, Dr. on idiopathic fever 225, 470
Jacquin, Prof. J. F. his "*Elements of Chemiftry*" 211
Jadelot, citz. J. F. N. his "*Defcription of an extraordinary human fkull*" 307
Jaeger, Dr. on phofphorus 299
Jenner, Dr. his enquiry into the caufes and effects of the cow-pox 2
Imhoff, on medical electricity 54
Inaugural differtations, American 88
Inoculation, controverfy on the fubject of, at Paris 202
Inventions, theories and fyftems of medicine, &c. during the eighteenth century 89
Journal, phyfical, of Gren, continuation of by Prof. Gilbert 203

Iron ore, difcovery of a new variety in America 202

Iron, the medicinal properties of 389

Kant and Hufeland, Prof. treatife on the art of prolonging life 196
——————, 496
Kaftelyn, on preferving mercurial preparations
King, Dr. on the datura ftramonium 278
Klipftein, Mr. his analyfis of quartz 515
Klaproth, Prof. his difcovery of vegetable alkali in mineral fubftances 300
Knee, cafe of fwelling of the, by Hufeland 393
Koelner, Dr. his publication of chemico-phyfiological effays 204
Kok, Dr. his "*Difcourfe on Medicine*" 415
——, his difcourfes on medicine 414
Krügelftein, Dr. on Typhus 394

Lacepede, citz. his "*Natural Hiftory of Fifhes*" 208
Laffecteur, citz. on the phyfical and moral difeafes of women 310
Lagrange, citz. Buillon, on the origin and properties of liquid ftyrax 166
——————, his manual of a courfe of chemiftry
Lampadius, Prof. on obtaining a new inflammable fluid from carbon 301
——, M. on the honey-ftone 399
Lamark's "New Theory of Chemiftry" 109
——, citz. on the new claffification of fhells 202
La Metherie, on a mineral alkali in the *Salfula Soda, Linn.* 301
Laquiere, Adrienne, citizenefs, on the Cæfarian operation 197
Lawrence, Mr. on the origin of the cow-pox 114
Lempriere, Mr. his "*Practical Obfervations*," &c. 409
Lent's "Differtation on Peftilential Vapours" 98
Lentin, Dr. his remarks on the cure of caries offium 294
Le Roy's new method of curing eryfiphelatous inflammations, &c. 84
Leroy, Prof. on the ufe of hot flour in eryfipelas, &c. 154
L'Heritier, citz. his prefentation of new plants the National Inftitute 201
Link, Prof. on the multiplied combinations of falts 301
Lowitz, Prof. his procefs of purifying molaffes
Lubbock, Dr. on the difcoveries of Mayow 220
——, Dr. on the publicity of Mayow's doctrines

Mai, Dr. his Latin oration at Heidelberg 88
Marcard, Dr. his advice to pregnant women in cafes of naufea and vomiting 284
Marfh, Mr. on the deleterious effects of the Lolium 423
Martin, Col. on deftroying the ftone in the bladder 120
Marum, Mr. on the temperature of the electric fluid 299
Mayow, account of, by Dr. Mitchill 298
Meale, Dr. on the digitalis purpurea 56
Memoirs of the French Medical Society 395
Memoirs, account of philanthropic 513
Michaelis, Dr. of Liepzig, his tranflation into German of Fordyce's "*Third Differtation on Fever*" 203
Miller, Dr. E. his remarks on the effects of abftinence 43
——, Dr. Ed. of New-York, his "*Inquiry concerning Cutaneous Perfpiration*" 287
Mineralogy, cabinets of 87
Mineral waters, citz. Goldfchmidt, on factitious 3[illegible]3
Mifcellaneous facts and remarks 283
Mitchill, Dr. his remarks on manures 45
——, Dr. on medical geography 254
——, and Miller, Drs. on carbon 279
Mönch, Mr. on gangrene and fphacelus 510
Monnet, citz. his "Demonftration of the Fallity of the Principles adopted by the Modern Chemifts" 98
Monftrofity, cafe of, by Mr. Pulley 224
Mons, citz. Van, on the purification of gumrefins 516
——————, on the means of purifying air in fick chambers 245
Morichau, Beauchamp, citz. on an affection of the heart 298
Morveau, cit. Guyton, on the means of difcovering the prefence of fulphuric acid 301
Mufk, artificial, recommended in the hooping-cough, by Prof. Hufeland 181

National Inftitute of France, their attention to learned foreigners 199
Nemnich, Mr. his nomenclator 513
——, his collection of phyfical works ib.

Odier, Dr. on the magifterium bifmuthi 512
Olivier, citz. his appointment to the Republican School, in zoology 304
Oxyd, yellow, experiments on its acid properties 239

Pallas's travels, a tranflation of, preparing for the prefs 403
Parkinfon, Mr. James, his "*Medical Admonitions*," &c. 310, 449
Parmentier and Deyeux, their "Phyfico-economical Library" 108
Pearfon, Dr. his enquiry concerning the cow-pox 5
——, Dr. his letter on cow-pox 113
Peck, Mr. on the new treatment of fteatomatous tumours 422
Philadelphia, Chemical Society of 85
Philofophical Society, American, their circular letter 87
Phofphorus, remarkable effects of 85
Phyficians, College of, proceedings of 106
Plants, collection of 97
Polidor, Dr. his "Memoirs refpecting the Cure of contagious Typhus Fever" 104
Prize-queftions propofed by the Phyf. Soc. of Guy's Hofpital 205
—————— of the Batavian Society, on the purification of water 296
—————— of the Lyceum Medicum Londinenfe 330
—— by the Medical Society of Paris 399

Prouft, citz. on the chemical combinations of tin 200

Publications, new medical, in April 311

———, new medical, in May 415

———, lift of new medical 214

Proctor, Mr. on the unguentum hydrargyri 356

Promotions, lift of medical, in the army 207

Propofal for improving the practice of medicine, by the introduction of chemical tables 180

——— for a new method of removing fteatomatous tumours 282

Pulley, Mr. on a cafe of monftrofity 224

Purton, Mr. on the cure of the croup 510

Quackery, on the moft effectual means of checking 337

R. B. M. on aneurifm of the aorta 331
Redfearn, Dr. on the diabetes mellitus 218
Remarks on the practice of furgery and phyfic in France 81
Rheumatic affections, remedies in 384
Richter, Dr. his works on the theories of chemiftry recommended 179
———, Dr. on the cryftallized acid of lemons 302
———, Dr. on acid of lemons 401
———, on the citrate of iron ib.
Ricketfon, Dr. his obfervations on the fpring months 79
Rollo, Dr. on "Cafes of the Diabetes Mellitus; with the Refults of the Trials of certain Acids, &c. in the Cure of the Lues Venerea" 102
Rumford, Count, his effays 80
Rufh, Dr. his "Medical Inquiries and Obfervations" 105

Salt of cryftals obtained by Van Mons, in the decompofition of fal-ammoniac 301
Scherer, Dr. his "Univerfal Chemical Journal" 82
———, Dr. on Canton's phofphorus 299
———, on carbon ibid.
Schmidt's "Chemical and Pharmaceutical Effays" 109
Schrank, Prof. on the phyfiology of vegetables 400
Seamen, prefervation of the health of 340
Selig, Dr. on fennel in confumption 384
Sheep, directions refpecting, &c. 107
Sherwin, Mr. his work on abforption 403
Simmons, Mr. W. "*On the Cæfarean Operation*" 309
——— on the "*Cæfarean Operation*" 410
Sims, Dr. John, his letter on the cow-pox, 12
———, Dr. on inoculation for the cow-pox 230
Smith's "Sketch of the Revolutions in Chemiftry" 99
———, Dr. I. C. "*On nitrous Vapours*" 213
———, Dr. I. C. a new edition of his work on the jail-diftemper 90
Sneyd, Mr. of Belmont, his method of preferving the feeds of plants 201
Society, Medical, of New-York, committee chofen to inveftigate the origin of diftempers 202
Spallanzani, Prof. on artificial impregnation, 47
———, the late abbé, his difcovery of a fpring of frefh water in the Mediterranean Sea 204
Sprengel's "Hiftory of Medicine" 83
St. Fond, tranflation of his "Travels in England, Scotland, and the Hebrides," &c. 90
———, his "Travels," &c. 414
Stratton, Dr. James, on omental hermia 158
Struve, Dr. his remedy for the hooping-cough 84
———, Dr. his "Manual for thofe Mothers who are concerned in the health of their offspring" 107
Sulphuric acid, difoxygenated by citizen Van Mons 300

Table, calculated for practice 152

——— of difeafes on board the Aftrea 347

Tape-worm, deftroyed by electricity 277

Tarbes, Roch, his "*Epitome of the Practice of Phlebotomy*" 309

———, on diftinct impregnation 399

Tennant, Mr. on arthritic concretions 197

Thilenius, Dr. on the aqua lauro cerafi 511

Thornton, Dr. his "Medical Extracts" 89

Thunberg, Prof. on the oil of cajeput 286

Tiffot, on "Dietetic Regimen, in the Cure of Difeafes" 106

Tode, Prof. on the gout, hypochondriafis, and hyfteria 149

———, Prof. his "Preliminaries of a Medical Peace" 169

Townfend, Mr. his "Phyfician's Vade Mecum" 89

Tranfactions of the Natural Hiftory Society of Copenhagen 297

Tranflations of Englifh Medical Works into German 402

Tromfdorff, Prof. on the cryftallization of lime 515

———, on the reduction of zircone 516

———, on the zoonic acid ib.

Trotter, Dr. his "Medicina Nautica" 80

———, ditto 407

Turton, Dr. W. his "*Syftema Naturæ*" 303

Valentin, citz. Louis, his anfwer to a query on inoculation 289

Vaughan, Dr. on the danger of introducing unknown poifons 450

Vauquelin and Fourcroy, citizens, on producing artificial cold 200

Vicq d'Azyr, his obfervations on practical medicine 395

Ulcers, on the treatment of inveterate 186

Underwood, Dr. his "Treatife on the Difeafes of Children, &c. 89

Unguentum hydrargyri, Mr. Proctor on the 356

Uterus, on the amputation of, by Baudeloque and Lamonier 392

Wainewright, Mr. on the danger and treatment of pins fwallowed 425

Walker, Mr. John, on a cafe of the cow-pox, 118

Walter, Dr. J. G. his "*Defcription of the Bones*" 306

Ward, Mr. on the application of opium by friction 441

Wardenburg, his letters of a phyſician 414

Warm bath, obſervations on, by Dr. Dewees 288

Webſter, Mr. his "Hiſtory of Peſtilential Diſeaſes" 79

Weikard, Dr. M. A. on the Chineſe remedy in lues 512

Wiegleb, M. his analyſis of the black chalk of Bayreuth 516

Werner, Prof. on the non-exiſtence of light 515

White, Mr. his "*Treatiſe on the Gradation of Man*" 205, 405

———, on gangrene and ſphacelus 509

Willich, Dr. his treatment of the ſame 510

———, on Anguſtura bark ibid.

Willich, on the treatment of biliary calculi 507

———, Dr. on the uſe of flannel next the ſkin 39

Wildenow, Prof. his deſcription of the Balm of Gilead 33

Willemet, P. R. his "*Mauritian Herbal*" 211

Woodville, Dr. on the progreſs of the cow-pox 417

Wright, Dr. W. his tranſlation of Coray's "Hippocrates on Air, Water, and Climates" 89

Wurzer, prof. on the means of procuring pure pot-aſh 300

Yeates, Dr. his letter on the treatment of pulmonary complaints 20

———, Dr. on the diſcoveries of Mayow 327

### *References to the Plates.*

Page.

I. A coloured Plate, exhibiting the Eruption of the *Cow-pox* on the Arm of a Milker (given with Number II.) deſcribed in No. I. - - - - - - - 1, 11.

II. A newly-invented Inſtrument for the Extraction of Teeth, by Dr. W. Dyce, (No. I.) - - - - - 13, 17.

III. The *Amyris Gileadenſis* deſcribed by Prof. Wildenow, (No. I. contains the Plate.) - - - - - 33, 39.

IV. An Engraving exhibiting the Proceſs of the Puſtules of the *Cow-pox* on Mr. Walker's Child, with a Deſcription by the Father, (the Plate was delivered with Number III.) 118, 120.

V. The Gazolitre of Cit. Goldschmidt, (No. IV.) deſcribed 367.

VI. An Apparatus for applying Gaſes to Wounds, by Dr. Rollo, (the Plate with IV.) deſcribed in No. V. - 431 & ſeq.

VII. Outline of a Plan of Military Hoſpitals, by Mr. Stewart Henderson, (in No. V.) deſcribed on the Plate, and referred to - - - - - - - - 461, 462.

